# Analysis of risk factors for delayed extubation after surgical anesthesia in obese patients undergoing abdominal laparoscopic surgery for gastrointestinal diseases: a single-center retrospective study

**DOI:** 10.3389/fcvm.2026.1875472

**Published:** 2026-08-20

**Authors:** Mengyuan Deng, Dexing Liu, Xiangyin Wei, Wanqiu Yu, Fushan Tang, Zhaoqiong Zhu

**Affiliations:** 1 Department of Anesthesiology, Affiliated Hospital of Zunyi Medical University, Zunyi, China; 2 Department of Clinical Pharmacy, Key Laboratory of Basic Pharmacology of Guizhou Province and School of Pharmacy, Zunyi Medical University, Zunyi, China; 3 Early Clinical Research Ward, Affiliated Hospital of Zunyi Medical University, Zunyi, China

**Keywords:** body mass index, delayed extubation, laparoscopic surgery, obesity, risk factors

## Abstract

**Background and aims:**

Delayed extubation of the endotracheal tube (DE) can occur after surgical anesthesia. There has been particular interest in DE among patients with obesity (defined as BMI ≥ 30 kg/m² according to World Health Organization criteria), but the risk factors remain unclear.

**Methods and results:**

We aimed to identify risk factors for DE after abdominal laparoscopic gastrointestinal surgery. The incidence of DE in this setting was approximately 10%. Through multiple logistic regression, we identified multiple risk factors for DE. In the overall gastrointestinal cohort, BMI and age showed the strongest correlation with DE, with a combined AUC of 0.875. The cutoff values for BMI (31.765) and age (53.5 years) represent risk-discrimination thresholds for identifying patients at higher risk of DE. In the bariatric subgroup, the factors that showed the strongest discriminatory ability were BMI, age, male sex, propofol dosage, sevoflurane dosage, and fasting blood glucose, with a combined AUC of 0.920. The exploratory cutoffs—BMI 36.32, age 41.5 years, male sex, propofol 107.5 mL, sevoflurane 35 mL, and fasting blood glucose 5.935 mmol/L—may help identify patients at relatively higher risk of DE.

**Conclusions:**

BMI and age are the most reliable predictors of DE in patients undergoing gastrointestinal surgery. In addition to BMI and age, other important factors include male sex, dosages of propofol and sevoflurane, and fasting glucose levels. For patients with obesity, the combined influence of these factors yielded an AUC of 0.920 for predicting delayed extubation.

## Introduction

Delayed extubation of the endotracheal tube (DE) refers to the failure to remove the tube within the anticipated timeframe following surgical anesthesia, often leading to the need for continued ventilator support. This delay can result in extended hospital stays and various postoperative complications, including airway injury, edema, pneumonia, and cardiac insufficiency. As a consequence, it can waste healthcare resources and significantly increase the cost of patient care (1). Therefore, it is vital to identify the risk factors associated with delayed extubation (2).

DE after anesthesia for gastrointestinal surgery is clinically complex (3). The risks associated with surgery are closely linked to the type and characteristics of the gastrointestinal procedure, the anesthesia process, and the patient's individual circumstances. For instance, gastrointestinal surgeries vary widely in type, patient age distribution, operation duration, and intraoperative blood loss (4). A considerable proportion of patients are middle-aged or older (aged 50–65 years) (5). Their nutritional status often indicates trends of both malnutrition and obesity, frequently associated with various comorbidities (6, 7). If delayed extubation after gastrointestinal surgery could be predicted, identifying high-risk surgery types and patient age would be crucial for managing the Post-Anesthetic Care Unit (PACU).

## Methods

### Definition

A predefined standardized definition of DE was established. Extubation was attempted only when the following clinical criteria were met simultaneously: adequate spontaneous breathing (tidal volume ≥ 6 mL/kg, respiratory rate 12–20/min), recovery of consciousness (eye opening or obeying commands), return of protective airway reflexes (swallowing and effective cough), hemodynamic stability (vital signs within ± 20% of baseline), and SpO₂ > 92%. DE was defined as failure to safely extubate within 15 min after the end of surgery, excluding cases with planned postoperative ventilation or deliberate sedation. This definition was applied uniformly to all cohorts. The duration of endotracheal intubation was defined separately as the total time from PACU admission to extubation.

### Data source

All data for the retrospective study were obtained from the hospital HIS information system (Haitai Medical Information System Co., Ltd., Nanjing, China) and the anesthesia information management system (Madiston Medical System Technology Co., Ltd., Suzhou, Jiangsu, China). Both of the medical information system and the anesthesia information management system belong to the common electronic medical record management system in Chinese hospitals, which can save complete medical documents.

### Inclusion and exclusion criteria for abdominal laparoscopic surgery for gastrointestinal diseases

**Figure 1:**
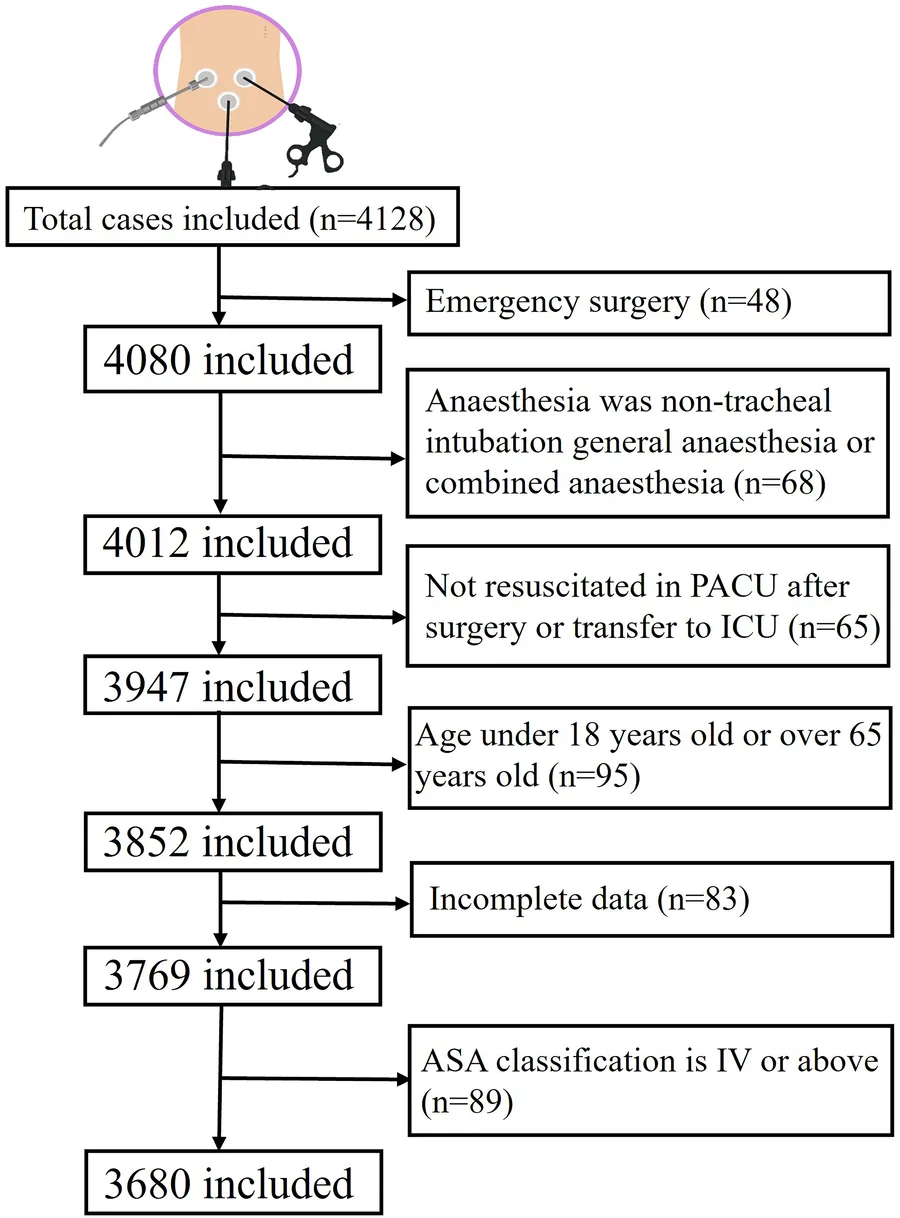
Flowchart of patient selection for the gastrointestinal cohort. A total of 4,128 cases were screened, with exclusions applied sequentially for emergency surgery, anesthesia type, resuscitation, age, incomplete data, and ASA classification, resulting in 3,680 included patients.

Patients who underwent elective abdominal laparoscopic surgery in the Department of Gastrointestinal Surgery at the Affiliated Hospital of Zunyi Medical University between January 1, 2021, and December 31, 2023, were included in this study. Their surgical anesthesia data were extracted from the HIS information system and the anesthesia information management system to analyze the factors influencing delayed extubation after surgical anesthesia. (Figure 1).

#### Criteria for inclusion (gastrointestinal diseases)

(1) Complete clinical data available; (2) Age between 18 and 65 years; (3) ASA classification II or III; (4) Elective surgical patients; (5) Endotracheal intubation performed in the operating room; (6) Anesthesia method: general anesthesia with endotracheal intubation; (7) Surgical procedures must be conducted in the gastrointestinal surgery department; (8) Postoperative recovery in the PACU; (9) Obesity defined as BMI ≥ 30 kg/m² according to World Health Organization criteria.

#### Criteria for exclusion (gastrointestinal diseases)

(1) Patients younger than 18 years or older than 65 years; (2) ASA classification of IV or higher; (3) Individuals undergoing emergency surgical procedures; (4) Patients who arrived in the operating room with an endotracheal tube in place; (5) General anesthesia administered through methods other than endotracheal intubation or the use of a combination of two or more anesthesia techniques; (6) Incomplete clinical data; (7) Failure to be admitted to the PACU for emergence at the end of the procedure.

### Inclusion and exclusion criteria for bariatric surgery (including sleeve gastrectomy and gastric bypass)

**Figure 2:**
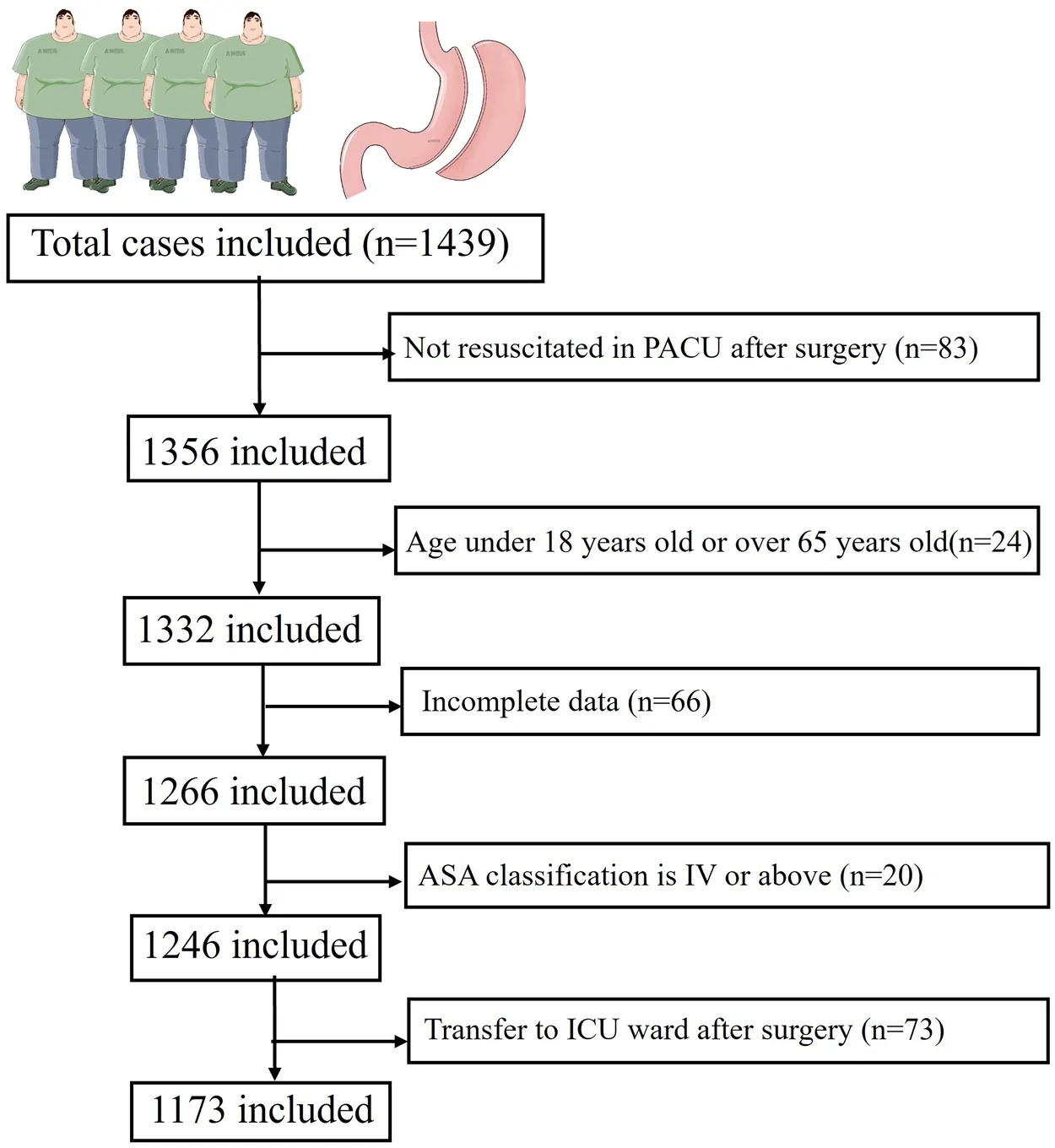
Flowchart of patient selection for the bariatric subgroup. From 1,439 initial cases, exclusions were made for resuscitation status, age, incomplete data, ASA classification, and ICU transfers, leaving 1,173 included patients.

Patients who underwent bariatric surgery in the Department of Gastrointestinal Surgery at the Affiliated Hospital of Zunyi Medical University from January 1, 2021, to December 31, 2023, were included in this study. Their surgical anesthesia data were extracted from the Hospital Information System (HIS) and the Anesthesia Information Management System (AIMS). Additionally, factors influencing delayed extubation were analyzed. (Figure 2).

#### Criteria for inclusion (bariatric surgery)

(1) Complete clinical data; (2) Age between 18 and 65 years; (3) ASA classification of II to III; (4) Elective surgical patients; (5) Patients who are intubated in the operating room; (6) Anesthesia method: general anesthesia with endotracheal intubation; (7) Type of surgery: bariatric surgery (sleeve gastrectomy or gastric bypass); (8) Complete clinical data (repeated); (9) Admission to the Post-Anesthesia Care Unit (PACU) for recovery after surgery; (10) Return to the ward after surgery.

#### Criteria for exclusion (bariatric surgery)

(1) Age: under 18 or over 65 years; (2) ASA classification of 4 or higher; (3) Emergency surgical patients; (4) Arrival with an endotracheal tube in place; (5) Use of non-tracheal intubation general anesthesia or a combination of multiple anesthesia techniques; (6) Lack of complete clinical information; (7) Inability to recover in the Post-Anesthesia Care Unit (PACU).

## Ethics approval

This retrospective study was conducted in line with the principles of the Declaration of Helsinki. It was approved by the Ethics Committee of the Affiliated Hospital of Zunyi Medical University (KLL-2024-112). The Ethics Committee waived the requirement for informed consent due to the anonymized nature of the data. Clinical trial: Not applicable.

### Variables

The study evaluated the effects of various factors on DE following gastrointestinal abdominal laparoscopy. These factors included gender, age, surgical grade, ASA classification, BMI classification (with obesity defined as BMI ≥ 30 kg/m² per WHO criteria), duration of anesthesia, duration of surgery, and dosages of anesthetic agents (sevoflurane, remifentanil, sufentanil, propofol, rocuronium bromide, and atracurium). Additionally, the impact of these factors on delayed extubation was specifically examined in patients undergoing bariatric surgery: (1) demographic characteristics and preoperative comorbidities: gender, age, BMI, corrected body weight (ABW), comorbidities (including obesity, diabetes mellitus, fatty liver disease, impaired glucose tolerance, insulin resistance, hyperlipidemia, hypertension, hyperuricemia, acanthosis nigricans, gastroesophageal reflux, mixed sleep apnea syndrome, fat excess, gout, hepatic insufficiency, hyperinsulinemia, coronary artery disease, chronic gastritis, duration of surgery, duration of anesthesia, and duration with tube). (2) Intraoperative medication, input and output: sevoflurane, sufentanil, remifentanil, propofol, rocuronium bromide, atracurium, infusion volume, and bleeding. (3) Tests: fasting blood glucose, glycosylated hemoglobin, triglycerides, high-density lipoprotein, and low-density lipoprotein. Surgery types in the gastrointestinal cohort include gastric resection, intestinal resection, and other abdominal procedures；the bariatric cohort only includes sleeve gastrectomy and gastric bypass. Anaesthetic drug dosages (propofol, sevoflurane, remifentanil, sufentanil, rocuronium bromide, and atracurium) were recorded as total intraoperative volumes (mL), as derived from the anaesthesia information system. Weight-standardized units (e.g., mg/kg or mg/kg/h) were not calculated due to the retrospective data constraints, which precluded reliable conversion.

## Statistical analysis

SPSS 29.0 (IBM Corp., Armonk, NY, USA) was employed for data organization and analysis. Measurement data that adhered to a normal distribution were presented as $\bar{x}\pm s$. In cases of equal variance, *post hoc* comparisons were carried out using the LSD test. For measurement data that did not conform to a normal distribution, the median and interquartile ranges were reported. The Kruskal–Wallis rank sum test was utilized for group comparisons, followed by the Bonferroni test for *post hoc* analyses. Count data were expressed as frequencies (percentages), with comparisons between groups conducted using the chi-square test or Fisher's exact test. Binary logistic regression analysis was performed for multifactorial analysis, and ROC curve analysis was employed to evaluate risk prediction performance. Variables were selected using a combined strategy based on univariate screening (***P*** ***<*** ***0.05***) and clinical relevance. Variables that did not reach the statistical threshold but were considered clinically important based on established literature and pathophysiological plausibility—such as anaesthetic drug dosages and key comorbidities—were also retained to ensure comprehensive confounder adjustment. For the two correlated time-related variables (duration of surgery and duration of anaesthesia), both were considered candidate predictors and were entered into the multivariable models (Table 2 for the overall gastrointestinal cohort and Table 5 for the bariatric subgroup). Drug dose analyses were performed using total intraoperative volumes. ***P*** ***<*** ***0.05*** was deemed statistically significant.

## Results

A total of 3,680 patients undergoing elective abdominal laparoscopic gastrointestinal surgery were included. Among these, 368 (9.9%) were in the DE group and 3,312 (90.1%) in the non-DE group. 15 factors were analyzed, and 11 confounding factors were ultimately identified.

Comparison of ASA classification, remifentanil dosage, sufentanil dosage, and atracurium dosage between the two groups showed no statistically significant difference. In the delayed extubation group, the proportion of gender “male” age, the proportion of surgical grade “4”, BMI, duration of anesthesia, duration of surgery, sevoflurane dosage, propofol dosage, rocuronium bromide dosage were statistically significant (***P*** ***<*** ***0.05***) (Table 1). All indicators with differences were included in the subsequent multifactorial logistic regression. The results showed that gender was “male” (OR: 2.92, 95% CI: 1.475–5.78, *P* = 0.002), age (OR: 1.241, 95% CI: 1.171–1.315, *P* *<* 0.001), BMI (OR: 1.428, 95% CI: 1.347–1.513, *P* *<* *0.001*), surgical grade 3 (OR: 8.024, 95% CI: 0.855–75.272, *P* = 0.068), surgical grade 4 (OR: 8.909, 95% CI: 1.169–67.916, *P* = 0.035), and duration of surgery (OR: 1.008, 95% CI: 1.003–1.013, *P* = 0.002), duration of anesthesia (OR: 1.009, 95% CI: 1.003–1.014, *P* = 0.002), sevoflurane (OR: 1.009 95% CI: 0.992–1.026, *P* = 0.292), the dosage of propofol (OR: 1.004, 95% CI: 0.997–1.011, *P* = 0.258), and the dosage of rocuronium bromide (OR: 1.006, 95% CI: 0.997–1.014, *P* = 0.185) (Table 2).

**Table 1:** Analysis of factors influencing delayed extubation in patients undergoing elective abdominal laparoscopic surgery.

Marker	No delayed (ND) (*n* = 3,312)	Delayed (D) (*n* = 368)	*P* value
Gender, *n*(%)			<0.001
Female	1,930 (58.0)	142 (38.6)	
Male	1,382 (41.7)	226 (61.4)	
Age (years)	44.00 (37.00, 50.00)	54.00 (47.00, 58.00)	<0.001
Surgical grade, *n* (%)			<0.001
II	1,184 (35.7)	111 (30.2)	
III	1,277 (38.6)	117 (31.8)	
IV	851 (25.7)	140 (38.0)	
ASA classification, *n* (%)			0.061
I	1,181 (35.7)	116 (31.5)	
II	1,968 (59.4)	187 (50.1)	
III	163 (4.9)	65 (17.7)	
BMI (kg/m^2^)	26.35 (24.22, 30.12)	32.86 (27.69, 38.01)	<0.001
Duration of anaesthesia (min)	110.00 (90.00, 162.00)	180.00 (120.00, 339.00)	<0.001
Duration of surgery (min)	80.00 (60.00, 120.00)	130.00 (85.00, 300.00)	<0.001
Sevoflurane (min)	0 (0, 30.00)	10.00 (0, 30.00)	0.043
Remifentanil (mg)	1.00 (1.00, 2.00)	1.00 (1.00, 2.00)	0.968
Sufentanil (*μ*g)	30.00 (25.00, 35.00)	30.00 (25.00, 35.00)	0.665
Propofol (mg)	50.00 (50.00, 80.00)	50.00 (50.00, 100.00)	0.012
Rocuronium bromide (mg)	50.00 (50.00, 70.00)	65.00 (50.00, 90.00)	0.002
Atracurium (mg)	0 (0, 0)	0 (0, 0)	0.933

Note: Drug dosages are presented as total intraoperative volumes (mL). Weight-standardized units were not calculated due to retrospective data limitations. Values with *P* < 0.001 were regarded as statistically significant.

**Table 2:** Multifactorial logistic regression for delayed extubation (multifactorial logistic regression analysis for elective abdominal lumpectomy).

Marker	*P*	OR	95% CI
Gender, *n*(%)			
Male	0.002	2.920	[1.475, 5.780]
Age (years)	<0.001	1.241	[1.171, 1.315]
BMI (kg/m)^2^	<0.001	1.428	[1.347, 1.513]
Surgical grade, *n* (%)			
III	0.068	8.024	[0.855, 75.272]
IV	0.035	8.909	[1.169, 67.916]
Duration of surgery (min)	0.002	1.008	[1.003, 1.013]
Duration of anaesthesia (min)	0.002	1.009	[1.003, 1.014]
Sevoflurane (min)	0.292	1.009	[0.992, 1.026]
Propofol (mg)	0.258	1.004	[0.997, 1.011]
Rocuronium bromide (mg)	0.185	1.006	[0.997, 1.014]

Note: Drug dosages are presented as total intraoperative volumes (mL). Weight-standardized units were not calculated due to retrospective data limitations. Values with *P* < 0.001 were regarded as statistically significant.

### Area under curve (AUC) of ROC and cut-off point

**Figure 3:**
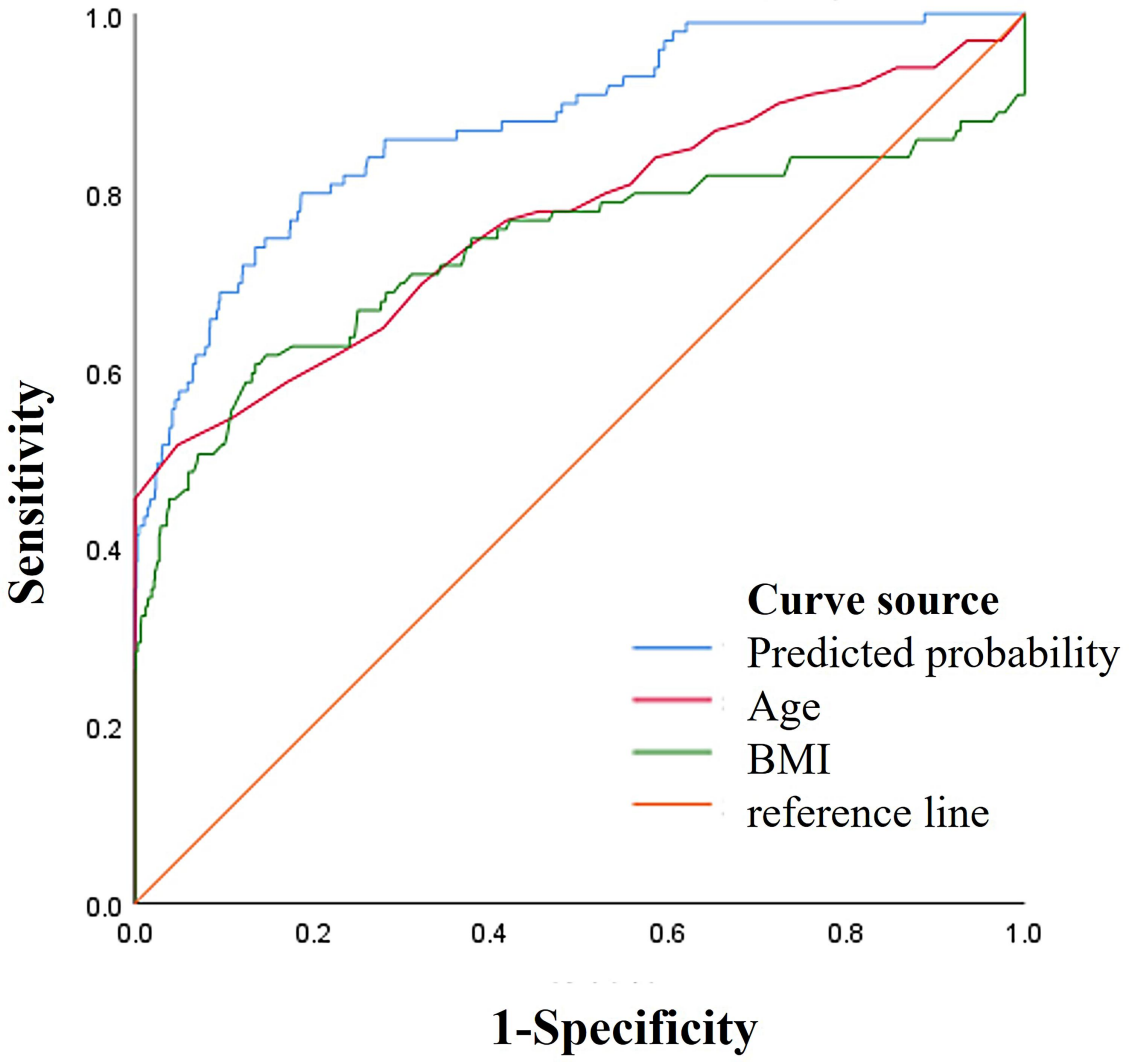
Receiver operating characteristic (ROC) curves for age, BMI, and their combination in predicting delayed extubation in the overall gastrointestinal cohort. The combined model achieved the highest AUC of 0.875.

The ability to discriminate between independent and combined factors (age, BMI, age and BMI combined) was assessed using ROC curves. Age (AUC: 0.768, 95% CI: 0.708–0.829, *P* < 0.001), BMI (AUC: 0.734, 95% CI: 0.663–0.804, *P* < 0.001), age and BMI combined (AUC: 0.875, 95% CI: 0.836–0.915, *P* < 0.001). The cut-off values for age and BMI were 53.5 and 31.765, respectively (Table 3). The combination of age and BMI shows better discriminative ability (Figure 3).

**Table 3:** Risk prediction efficacy analysis of BMI and Age for delayed extubation.

Marker	Cut-off	AUC	95% CI	*P* value
BMI	31.765	0.734	[0.663, 0.804]	<0.001
Age	53.5	0.768	[0.708, 0.829]	<0.001
Joint prediction	—	0.875	[0.836, 0.915]	<0.001

Note: Drug dosages are presented as total intraoperative volumes (mL). Weight-standardized units were not calculated due to retrospective data limitations. Values with *P* < 0.001 were regarded as statistically significant.

The bariatric subgroup included 1,173 patients, of whom 93 (7.9%) were in the DE group and 1,080 (92.1%) in the non-DE group. 39 factors were analyzed, and 15 confounding factors were ultimately identified.

Comparison of general conditions and preoperative comorbidities between the two groups: BMI, age, male, comorbid obesity, comorbid fatty liver, duration of surgery, duration of anesthesia, and duration with the tube in the delayed group were statistically different from those in the non-delayed group (*P* *<* 0.05). ABW, diabetes mellitus, impaired glucose tolerance, insulin resistance, hyperlipidemia, hypertension, hyperuricemia, acanthosis nigricans, Gastroesophageal Reflux, Mixed Sleep Apnea Syndrome, Hyperlipidemia, Cough Variant Asthma, Gout, Hepatic Insufficiency, Hyperinsulinemia, Coronary Artery Disease, and Chronic Gastritis were not statistically significant. Comparison of intraoperative medication, fluid intake, and output, and other surgical information between the two groups of patients: Sevoflurane dosage, Propofol dosage, and Rocuronium bromide dosage in the delayed group were statistically significant when compared with those in the non-delayed group (*P* *<* 0.05). In comparison of Sufentanil, Remifentanil, Atracurium, infusion volume, and bleeding volume, the difference was not statistically significant. Comparison of inspection indicators between the two groups of patients: the difference between fasting blood glucose in the delayed group and the non-delayed group was statistically significant (*P* *<* 0.05). In comparison of glycated hemoglobin, triglycerides, high-density lipoprotein, and low-density lipoprotein between the two groups, the difference was not statistically significant (Table 4). All the indicators of differences were included in the subsequent multifactorial logistic regression, which showed that gender was male (OR:2.653, 95% CI: 1.503–4.683, *P* *<* 0.001), age (OR: 1.125, 95% CI: 1.088–1.164, *P* *<* 0.001), BMI (OR: 1.256. 95% CI: 1.172–1.347, *P* *<* *0.001*), comorbid obesity (OR: 5.123, 95% CI: 0.945–27.777, *P* = 0.058), fatty liver (OR: 1.72, 95% CI: 0.739–4.004, *P* = 0.208), and duration of surgery (OR: 0.997, 95% CI: 0.991–1.003, *P* = 0.353), duration of anesthesia (OR: 0.994, 95% CI: 0.987–1.008, *P* = 0.063), sevoflurane dosage (OR: 1.021, 95% CI: 1.004–1.038, *P* *=* *0.013*), propofol dosage (OR: 1.01, 95% CI: 1.004–1.016, *P* *<* *0.001*), rocuronium bromide dosage (OR: 1.004, 95% CI: 0.997–1.01, *P* = 0.241), and fasting blood glucose (OR: 1.107, 95% CI: 1.038–1.182, *P* *=* *0.002*) (Table 5).

**Table 4:** Delayed versus non-delayed extubation group in bariatric surgery (comparison of demographic characteristics between delayed and non-delayed extubation groups in bariatric surgery).

Marker	No delayed (ND) (*n* = 1,080)	Delayed (D) (*n* = 93)	*P* value
Genders, *n* (%)			<0.001
Female	849 (78.6)	41 (44.1)	
Male	231 (21.4)	52 (55.9)	
Age (years)	33.00 (28.00, 39.00)	43.00 (34.00, 52.00)	<0.001
BMI (kg/m^2^)	33.98 (31.25, 37.18)	37.50 (35.28, 40.57)	<0.001
ABW	69.80 (64.40, 77.00)	70.80 (63.80, 77.20)	0.812
Preoperative comorbidities (*n*,%)			
Obesity	993 (91.9)	91 (97.8)	0.039
Diabetes	148 (13.7)	13 (14.0)	0.941
Fatty liver	869 (80.5)	83 (89.2)	0.038
Impaired glucose tolerance	22 (2.0)	5 (5.4)	0.089
Insulin resistance	467 (43.2)	34 (36.6)	0.211
Hyperlipidaemia	299 (27.7)	18 (19.4)	0.083
High blood pressure	178 (16.5)	20 (21.5)	0.215
Hyperuricaemia	279 (25.8)	21 (22.6)	0.490
Acanthosis nigricans	436 (40.4)	29 (31.2)	0.082
Gastroesophageal reflux	7 (0.6)	0 (0)	0.939
Mixed sleep apnoea syndrome	713 (66.0)	56 (60.2)	0.258
Hyperliposis	426 (39.4)	29 (31.2)	0.117
Gout	29 (2.7)	2 (2.2)	0.800
Liver insufficiency	8 (0.7)	1 (1.1)	0.800
Hyperinsulinaemia	23 (2.1)	4 (4.3)	0.327
Coronary heart disease	3 (0.3)	0 (0)	0.800
Chronic gastritis	21 (1.9)	2 (2.2)	0.800
Duration of surgery (min)	75.00 (65.00, 90.00)	80.00 (70.00, 100.00)	0.006
Duration of anaesthesia (min)	105.0 (95.00, 125.00)	120.00 (100.00, 184.50)	<0.001
Sevoflurane (mL)	10.00 (0, 20.00)	10.00 (0, 30.00)	0.002
Sufentanil (ug)	40.00 (30.00, 50.00)	40.00 (30.00, 50.00)	0.827
Remifentanil (mg)	1.00 (1.00, 1.00)	1.00 (1.00, 1.00)	0.782
Propofol (mL)	50.00 (50.00, 70.00)	70.00 (50.00, 120.00)	<0.001
Rocuronium bromide (mg)	80.00 (50.00, 100.00)	90.00 (70.00, 117.50)	0.014
Atracurium (mg)	0 (0, 0)	0 (0, 0)	0.880
Infusion volume (mL)	600.00 (600.00, 1,100.00)	700.00 (600.00, 1,100.00)	0.405
Bleeding volume (mL)	10.00 (10.00, 20.00)	10.00 (10.00, 20.00)	0.115
Fasting Blood Sugar	5.24 (4.69, 6.20)	5.52 (4.80, 7.72)	0.041
Glycated haemoglobin	5.70 (5.40, 6.20)	5.75 (5.40, 6.45)	0.528
Triglycerides	2.18 (1.43, 3.12)	2.02 (1.36, 3.24)	0.796
Glycated haemoglobin (GHG)	1.13 (0.99, 1.27)	1.12 (0.99, 1.33)	0.928
Low Density Lipoprotein	3.20 (2.78, 3.67)	3.26 (2.79, 3.63)	0.932

Note: Drug dosages are presented as total intraoperative volumes (mL). Weight-standardized units were not calculated due to retrospective data limitations. Values with *P* < 0.001 were regarded as statistically significant.

**Table 5:** Multifactorial logistic regression for delayed extubation (multifactorial logistic regression in weight loss surgery).

Marker	*P* value	OR value	95% CI
Male	<0.001	2.653	[1.503, 4.683]
Age (years)	<0.001	1.125	[1.088,1.164]
BMI (kg/m^2^)	<0.001	1.256	[1.172,1.347]
Obesity			
Yes	0.058	5.123	[0.945, 27.777]
Fatty liver			
Yes	0.208	1.720	[0.739, 4.004]
Duration of surgery (min)	0.353	0.997	[0.991, 1.003]
Duration of anaesthesia (min)	0.063	0.994	[0.987, 1.008]
Sevoflurane (min)	0.013	1.021	[1.004, 1.038]
Propofol (mg)	0.001	1.01	[1.004, 1.016]
Rocuronium bromide (mg)	0.241	1.004	[0.997, 1.010]
Fasting Blood Glucose	0.002	1.107	[1.038, 1.182]

Note: Drug dosages are presented as total intraoperative volumes (mL). Weight-standardized units were not calculated due to retrospective data limitations. Values with *P* < 0.001 were regarded as statistically significant.

### Area under curve (AUC) of ROC and cut-off point

**Figure 4:**
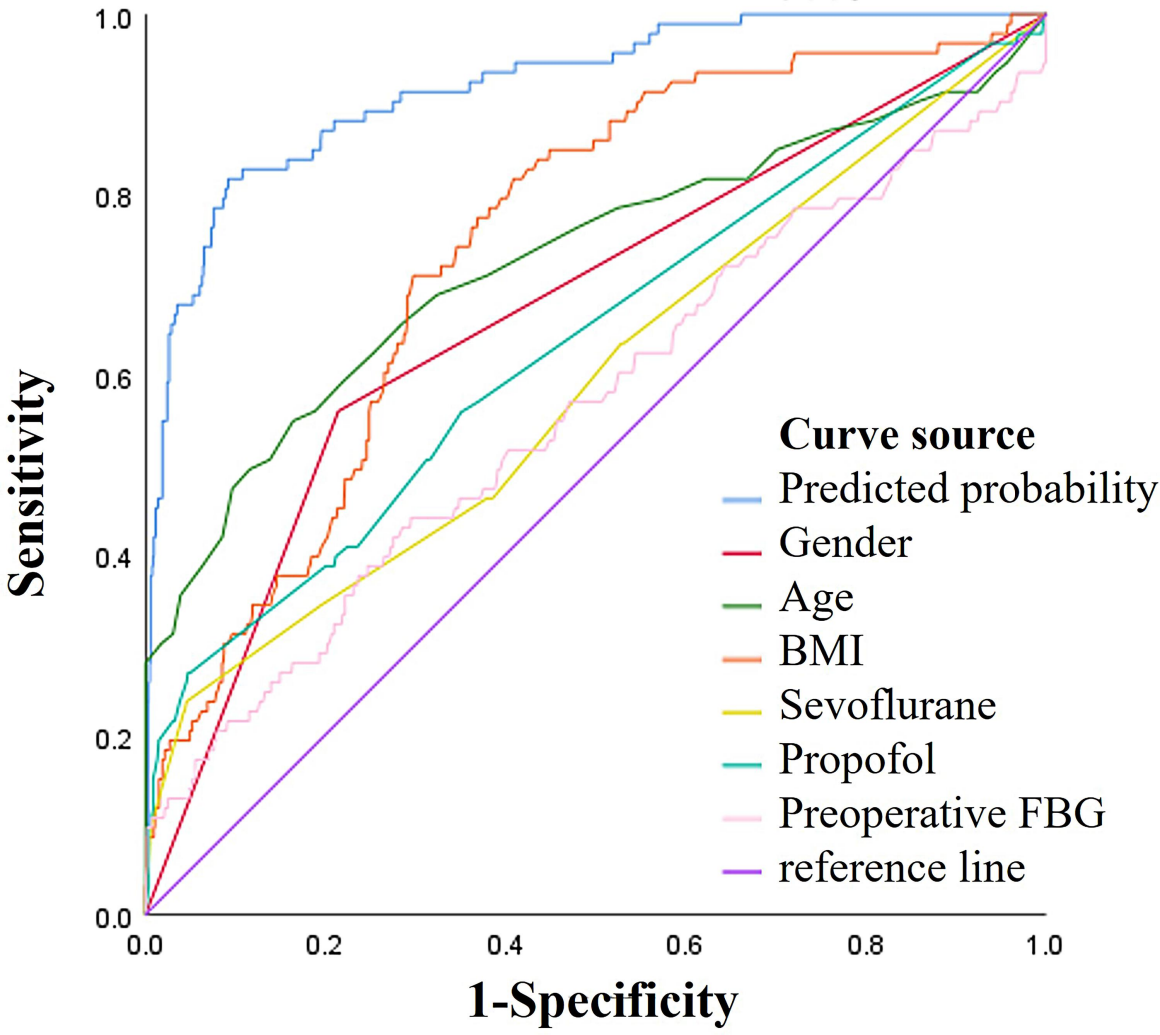
ROC curves for individual predictors (BMI, age, sex, propofol dosage, sevoflurane dosage, and fasting blood glucose) and their combined model in the bariatric subgroup. The combined model yielded an AUC of 0.920.

Independent vs. co-factors (BMI, age, sex, propofol, sevoflurane, preoperative fasting glucose, and co-prediction) were assessed using ROC curves. BMI (AUC: 0.743, 95% CI: 0.694–0.791, *P* < 0.001), age (AUC: 0.728, 95% CI: 0.662–0.794, *P* < 0.001), sex (AUC: 0.673, 95% CI: 0.611–0.734, *P* < 0.001), propofol (AUC: 0.636, 95% CI: 0.571–0.701, *P* < 0.001), sevoflurane (AUC: 0.591, 95% CI: 0.524–0.657, *P* *=* *0.004*), and preoperative fasting glucose (AUC: 0.564. 95%CI: 0.496–0.632, *P* *=* *0.041*) and combined prediction (AUC: 0.92, 95%CI: 0.889–0.95, *P* *<* *0.001*) (Tables 5, 6). The exploratory cut-off values derived from the ROC analysis were 36.32 for BMI, 41.5 years for age, 107.5 mL for propofol, 35 mL for sevoflurane, and 5.935 mmol/L for fasting blood glucose, with the combined model yielding an AUC of 0.920 (Figure 4).

**Table 6:** Risk prediction efficacy analysis of delayed extubation (risk prediction efficacy analysis of both groups of patients in bariatric surgery).

Marker	Cut-off	AUC	95% CI	*P* value
Tracheal intubation time (min)	164.50	0.868	[0.838, 0.899]	<0.001
BMI	36.32	0.743	[0.694, 0.791]	<0.001
Age (years)	41.50	0.728	[0.662, 0.794]	<0.001
Genders	—	0.673	[0.611, 0.734]	<0.001
Propofol (mg)	107.50	0.636	[0.571, 0.701]	<0.001
Sevoflurane (min)	35	0.591	[0.524, 0.657]	0.004
Fasting Blood Sugar	5.935	0.564	[0.496, 0.632]	0.041
Joint prediction	—	0.920	[0.889, 0.95]	<0.001

Note: Drug dosages are presented as total intraoperative volumes (mL). Weight-standardized units were not calculated due to retrospective data limitations. Values with *P* < 0.001 were regarded as statistically significant.

## Discussion

Obesity, defined as a BMI ≥ 30 kg/m² according to the World Health Organization criteria, is a global health crisis and a significant risk factor for multiorgan pathologies including cardiovascular, respiratory, digestive, and neurological disorders. Delayed extubation refers to the failure to remove the endotracheal tube within the anticipated timeframe following general anesthesia, primarily due to inadequate recovery of spontaneous ventilation and compromised airway protective reflexes. Generally, extubation delay is defined as extubation time exceeding 15 min. This threshold, established through statistical analysis of numerous studies and clinical practice, should be assessed in conjunction with the patient's spontaneous breathing recovery, cough reflex, swallowing reflex, and airway protection capability. Compared with normal-weight patients, obese patients are more susceptible to delayed extubation following general anesthesia for numerous reasons, resulting in severe consequences, and no satisfactory solution currently exists (the relationship between obesity and delayed extubation has been comprehensively reviewed, as shown in Table 7).

**Table 7:** Literature review on delayed extubation after general anesthesia in obese patients.

Section	Details
1. Obesity (5–8)	**1.1 Definition and Epidemiology:** Obesity is a prevalent global issue that is closely associated with more diseases, and its rates continue to climb. According to the World Health Organization (WHO) standards, adult with the BMI ≥30 kg/m^2^ is classified as obese.
	**1.2 Physiological Mechanisms:** – Cardiovascular System: Increasing the burden on heart, resulting in hypertension, coronary heart disease, and other conditions. – Respiratory System: Individuals are susceptible to sleep apnea-hypopnea syndrome (OSAHS) and decreased lung function, among others. – Digestive System: Triggers conditions such as non-alcoholic fatty liver disease. – Endocrine System: Insulin resistance, endocrine disruptions, and other issues. – Neurological System: Linked to an elevated risk of cognitive impairment and neurodegenerative diseases.
	**1.3 The Relationship Between Obesity and Delayed Extubation:** Due to the unique upper airway anatomy (e.g., excessive fat deposition in the neck), obese patients are more prone to airway obstruction. The anatomical peculiarity not only prolong the timing of extubation, but also can lead to post-extubation complications, such as upper airway obstruction and hypoxemia.
	**1.4 The Relationship Between Obesity and Neurocognitive Disorders:** Obesity may alter brain structure and function, for example, by causing the reduction in hippocampal volume, potentially leading to a decline in neurocognitive function. The metabolic disturbances and inflammation associated with obesity, may have detrimental effects on the brain, increasing the risk of neurocognitive disorders, such as Alzheimer's disease.
2. Delay in tracheal extubation after general anaesthesia surgery (2, 3, 4, 19, 20)	**2.1 Definition and Assessment:** Delayed extubation refers to the situation where, after the end of general anesthesia, a patient fails to recover sufficient spontaneous breathing and airway protective abilities within the expected time frame, thus making it impossible to remove the endotracheal tube in a timely manner. Delayed extubation is generally defined as taking more than 15 min, a threshold determined by statistical analysis from numerous studies and clinical practice. However, the assessment should take into account the patient's recovery of spontaneous breathing, cough and swallowing reflexes, and airway protective abilities.
	**2.2 Risk factors:** – Patient-related factors: Obesity, age, gender, etc. – Surgery-related factors: Surgery type, duration, and position. – Anaesthesia-related factors: Anaesthetic agents, dosages, and intraoperative management. – Consequences of delayed extubation: Prolonged hospital stay and increased medical costs; Increasing risk of respiratory infections and pulmonary complications; Increasing patient anxiety and discomfort; Impacting on systemic functions, e.g., increased burden on the circulation.
	**2.3 Management Strategies:** – Preoperative: Conducting a comprehensive assessment of respiratory function, airway status, and comorbidities. – Intraoperative: Selecting anaesthetic appropriately, optimize the anesthesia plan, and pay attention to drug dosages and metabolic characteristics. – Postoperative: Closely monitor respiratory status, oxygen saturation, and airway patency. Promptly address causes of delayed extubation, such as providing respiratory support, initiating anti-infection therapy, and relieving airway obstruction. For high-risk groups like obese patients, develop personalized anesthesia and extubation plans.
3. The link between obesity and delayed extubation after general anaesthesia (14–18)	Obesity can impact brain health, and raise the risk of neurocognitive diseases through mechanisms like metabolic disorders, chronic inflammation and insulin resistance. Delayed extubation after surgery can lead to hypoxia and alterations in cerebral hemodynamics, causing brain injury, potentially hindering the recovery of neurocognitive function, and may be associated with postoperative cognitive dysfunction (POCD) and other related conditions. In obese patients, physiological changes from delayed extubation after general anaesthesia may exacerbate neurocognitive function damage, elevate the risk of neurocognitive diseases, or worsen existing conditions.
4. Summary	Future could focus on three main areas: (1) Obesity management and recovery: Developing perioperative management strategies for obese patients, in order to enhance recovery and reduce delayed extubation. (2) Early intervention for neurocognitive diseases: Investigate ways to protect neurocognitive function in obese patients with delayed extubation, such as medications and cognitive training. (3) Multidisciplinary collaboration: Combine Endocrinology, Anaesthesiology, Neuroscience, etc., to better understand the relationship between obesity, delayed extubation, and neurocognitive diseases

In clinical work, we found that delayed extubation occurs after anesthesia for gastrointestinal surgery, the patient's age distribution is wide, comorbidities are many, and the types of surgery are complex. Suppose we can initially screen the patient's condition before surgical anesthesia. In that case, it will help us to make a preliminary determination of whether delayed extubation occurs after surgical anesthesia for the patient, to adjust the amount of anesthesia medication to make the patient go through the recovery period of anesthesia smoothly, to reduce the time of the respiratory machine with the tube and to reduce the incidence of pulmonary complications. Therefore, one of the objectives was to first focus on abdominal laparoscopic surgery in gastrointestinal surgery. The analysis showed that BMI and age correlate the highest in predicting delayed extubation. Their combined AUC reached 0.875, which implied that patients with gastrointestinal surgery, patients with higher BMI, and older patients are more likely to have delayed extubation after surgical anesthesia. The combined model yielded an AUC of 0.875, with BMI (31.765) and age (53.5 years) serving as risk-discrimination thresholds, BMI serves as an indicator of obesity. Obesity serves as an independent risk factor for the development of diseases, including hypertension & coronary heart disease, dyslipidemia&diabetes, obstructive sleep apnea-hypopnea syndrome (OSAHS) & respiratory failure, mild cognitive impairment (MCI) & Alzheimer's disease (AD) (8), imposing the heavy burden on obese patients, the healthcare system, and society at large.

In addition to BMI and age, the delayed group of patients undergoing abdominal lumpectomy in gastrointestinal surgery was compared with the non-delayed group. Significant differences were found in tracheal intubation length, gender, Propofol dosage, Sevoflurane dosage, and preoperative fasting blood glucose. Longer surgical duration is associated with increased intraoperative complexity and higher anaesthetic exposure, which may contribute to a rise in both intraoperative and postoperative complications, and is associated with delayed extubation (9). The influence of gender on delayed extubation may be partly attributable to biological differences. For example, elevated levels of free testosterone are closely associated with weight loss, as well as ultrasensitive C-reactive protein and leptin levels. Furthermore, fluctuations in postoperative testosterone levels are correlated with both weight loss and lipoinflammation. These factors may clarify the gender differences in delayed extubation following anesthesia for gastrointestinal surgery (10). Our analyses showed that propofol and sevoflurane dosages were significantly associated with DE. However, as these drug dosages may correlate with surgical duration—itself a known risk factor for prolonged recovery—the observed associations should be interpreted with caution and viewed as correlations rather than causal effects. These findings are consistent with previous reports on propofol pharmacokinetics in obese patients (11) and sevoflurane-related postoperative agitation (12). One possible explanation is that sevoflurane is associated with a higher incidence of postoperative agitation. This agitation may affect recovery quality and be related to longer recovery time. Our analysis also demonstrated a significant association between preoperative fasting blood glucose levels and DE. This may be partly explained by glucose fluctuations associated with neurocognitive recovery (13).

After evaluating the risk factors for delayed extubation in patients undergoing abdominal laparoscopic surgery in the realm of gastrointestinal surgery, we identified body mass index (BMI) and age as the most significant variables correlated with delayed extubation following surgical anesthesia. Consequently, we decided to focus our subsequent study on obese patients undergoing bariatric surgery and conducted a more in-depth analysis of the risk factors associated with delayed extubation in this population.

In obese patients who underwent bariatric surgery (including sleeve gastrectomy and gastric bypass), those in the non-delayed extubation group had lower proportions of males, body mass index (BMI), age, combined obesity, combined fatty liver, and shorter durations of surgery, anesthesia, and tube placement compared to the delayed extubation group. Both BMI and age are significant risk factors for delayed extubation in these patients. A study by Ma et al. indicated that the prevalence of Obstructive Sleep Apnea Syndrome (OSAS) was higher among male patients. This increased prevalence is primarily due to the larger cross-sectional area of the epiglottis and longer length of the oropharyngeal airway in males, which can lead to airway collapse and worsen the symptoms of OSA. This might explain the higher incidence of delayed extubation among obese male patients (14, 15). In addition to, with an increase in BMI, the proportion of comorbid morbid obesity and nonalcoholic fatty liver disease (NAFLD) gradually increases, which increases the risk of perioperative management. NAFLD is associated with hepatic insufficiency, which may affect coagulation and may be associated with delayed postoperative extubation; Secondly, NAFLD is closely related to fatty liver, which may exacerbate insulin resistance and increases the risk of cardiovascular disease; finally, adipose tissue accumulation may eventually develop into cirrhosis, decrease liver function in patients with obesity, and cause metabolic disease leading to delayed extubation, while adipose tissue accumulation may develop systemic inflammatory lesions, increase oxygen consumption in the brain, and aggravate preoperative anxiety and depression (16). The preoperative fasting blood glucose significantly impacts preoperative blood glucose levels. Preoperative fasting blood glucose may be associated with postoperative neurocognitive recovery, and fluctuations in blood glucose levels may be related to a decline in the rate of postoperative cognitive recovery in nondiabetic patients, and some studies have confirmed that non-diabetic patients may have a higher sensitivity to changes in blood glucose (17). Diabetic patients, given their disordered glucose metabolism, may be at increased risk of brain injury during surgery, trauma, and stress. Thus, preoperative glycemic control may be associated with cerebrovascular and neurodegenerative outcomes, which may in turn be related to delayed extubation in the postoperative period (18).

Our study examined delayed extubation in gastrointestinal surgery involving abdominal laparoscopy and bariatric surgery. BMI demonstrated good discriminatory ability, with AUCs of 0.734 (overall gastrointestinal cohort, Table 3) and 0.743 (bariatric subgroup, Table 6), respectively. When BMI is combined with factors such as the duration of intubation, age, propofol dosage, sevoflurane dosage, and fasting blood glucose levels, the combined AUC increased to 0.920. This suggests that male obese patients with older age, higher BMI, longer surgery duration, and higher dosages of propofol and sevoflurane face an increased likelihood of experiencing delayed extubation in the postoperative period. This finding carries important implications for anesthesiologists managing obese patients, as this population is at higher risk for delayed extubation during the recovery phase. Consequently, the incidence of adverse events following surgical anesthesia is elevated in this group, especially when they undergo more complex surgeries with longer operative times.

Our study primarily examines gastrointestinal surgery. Our analysis identified BMI (Body Mass Index) and age as the primary factors contributing to delayed extubation. To implement an “initial screening” strategy for gastrointestinal surgery, we focused on the patient demographic most closely associated with elevated BMI—specifically, obese patients. We then investigated the factors affecting delayed extubation within this group and confirmed that BMI remains a significant influence. Consequently, obesity consistently presents a risk for delayed extubation in gastrointestinal procedures. Therefore, during the anesthesia of obese patients, it is essential to manage both the dosage and type of medications used meticulously. By rationalizing drug administration, we can mitigate the metabolic burden on these individuals and diminish the chances of delayed extubation.

Delayed extubation is defined as the extended application of mechanical ventilation following surgical anesthesia. This practice is associated with an increased risk of postoperative complications, including acute kidney injury and delirium. Notably, studies have indicated that the in-hospital mortality rates for patients requiring prolonged mechanical ventilation can be as high as 50.3% (19). Furthermore, the extended duration of mechanical ventilation following surgical anesthesia places a significant burden on bed availability in the Post-Anesthesia Care Unit (PACU) and influences the distribution of anesthesiologists (20). Consequently, early identification of patients at high risk for delayed extubation is essential for enhancing patient outcomes and optimizing healthcare resource allocation. In our study, we examined the risk factors associated with delayed extubation in patients undergoing gastrointestinal surgery. We then concentrated specifically on obese patients, who are particularly impacted by these factors, thereby creating a “general-local” study. This “general-local” assessment model supports anesthesiologists in adopting a perspective that moves from “surgical department to surgery type to individual patient.”

The study does have several limitations. First, it is a single-center study with a limited sample size. Second, as with many retrospective studies, there are potential biases, including selection bias and information bias. Additionally, establishing causality is challenging because retrospective studies are observational and cannot randomly assign subjects. This raises the possibility that the study's results might be influenced by unknown or unmeasured confounding variables. Lastly, these studies may suffer from missing or inaccurately recorded data, which can undermine the reliability of the findings. Furthermore, if data is collected over a long period, it may be affected by technological changes, shifts in diagnostic criteria, and other factors. To address these limitations in future research, it is crucial to apply strict inclusion and exclusion criteria to ensure the reliability of the results.

Additionally, anaesthetic drug dosages were analysed as total intraoperative volumes rather than weight-normalized units (e.g., mg/kg), as the retrospective data lacked the granularity for reliable conversion. This should be considered when interpreting the dose-related findings. Furthermore, as a retrospective study, our findings demonstrate associations rather than causality, and residual confounding—particularly the correlation between drug dosages and surgical duration—cannot be excluded. Further external validation, model calibration assessments, and standardized metrics are warranted in future multicenter prospective studies.

## Conclusions

The results of our study indicate that combining BMI and age can effectively assess the likelihood of DE following surgical anesthesia. In the context of bariatric surgery, BMI serves as an independent risk factor for predicting DE in obese patients. When combining age, male sex, propofol dosage, sevoflurane dosage, and fasting blood glucose, the best predictors for DE were BMI and these other variables, achieving a combined AUC of 0.920. These findings should be interpreted as associations rather than causal relationships, pending prospective validation.

## Data Availability

The original contributions presented in the study are included in the article/Supplementary Material, further inquiries can be directed to the corresponding author/s.
